# Deletion of FGF9 in GABAergic neurons causes epilepsy

**DOI:** 10.1038/s41419-021-03478-1

**Published:** 2021-02-19

**Authors:** Moran Guo, Can Cui, Xueqin Song, Lijing Jia, Duan Li, Xiuli Wang, Hui Dong, Yanqin Ma, Yaling Liu, Zhiqiang Cui, Le Yi, Zhongyao Li, Yue Bi, Yuanyuan Li, Yakun Liu, Weisong Duan, Chunyan Li

**Affiliations:** 1 Department of Neurology, Second Hospital of Hebei Medical University, Shijiazhuang, Hebei 050000 China; 2 Neurological Laboratory of Hebei Province, Shijiazhuang, Hebei 050000 China; 3 Jiangsu Nhwa Pharm. Co. Ltd, Nantong, Jiangsu 210000 China; 4 Hebei General Hospital, Shijiazhuang, Hebei 050000 China

**Keywords:** Epilepsy, Molecular neuroscience

## Abstract

Fibroblast growth factor 9 (FGF9) has long been assumed to modulate multiple biological processes, yet very little is known about the impact of FGF9 on neurodevelopment. Herein, we found that loss of Fgf9 in olig1 progenitor cells induced epilepsy in mice, with pathological changes in the cortex. Then depleting Fgf9 in different neural populations revealed that epilepsy was associated with GABAergic neurons. Fgf9 CKO in GABAergic neuron (CKO^VGAT^) mice exhibited not only the most severe seizures, but also the most severe growth retardation and highest mortality. Fgf9 deletion in CKO^VGAT^ mice caused neuronal apoptosis and decreased GABA expression, leading to a GABA/Glu imbalance and epilepsy. The adenylate cyclase/cyclic AMP and ERK signaling pathways were activated in this process. Recombinant FGF9 proteoliposomes could significantly decrease the number of seizures. Furthermore, the decrease of FGF9 was commonly observed in serum of epileptic patients, especially those with focal seizures. Thus, FGF9 plays essential roles in GABAergic neuron survival and epilepsy pathology, which could serve as a new target for the treatment of epilepsy.

## Introduction

Epilepsy is one of the most common and complex neurological disorders, affecting approximately 65 million people around the world. Although researchers have striven to identify some genetic aspects of epilepsy, the underlying mechanism remains elusive^1^. Currently, the study of epileptogenesis focuses on the cellular and molecular alterations caused by pathogenetic events, including brain injuries and genetic alterations^2^. In the mammalian central nervous system (CNS), glutamate (Glu) and γ-aminobutyric acid (GABA) are considered the major excitatory and inhibitory neurotransmitter, respectively^3^. Both transmitters ensure normal neural circuit functionality, and a disrupted neurotransmitter GABA/Glu balance is associated with the pathophysiology of epilepsy^4,5^.

Fibroblast growth factors (FGFs) are a large family of growth factors that participate in the proliferation, differentiation, and migration of a variety of cell types. Recently, several studies suggested new roles of FGF members in epileptogenesis. Local delivery of FGF2 into the damaged hippocampus of mice could promote neurogenesis, reduce epileptogenesis, and partially save neuronal damage^6^. FGF7 and FGF22 promote the differentiation of GABA-mediated and Glu-mediated synapses, and are resistant or prone to epileptic seizures, respectively^7,8^.

One family member, FGF9 has been shown to alter the fate of neural progenitor cells by increasing proliferation, inhibiting astrocyte differentiation, and decreasing oligodendrocyte differentiation^9^. Fortin et al. reported that FGF9 did not affect the proliferation of oligodendrocyte progenitor cells, and only promotes the growth of mature oligodendrocytes without affecting myelin protein expression^10^. FGF9 is widely expressed in the CNS, but very little is known about the impact of FGF9 on epilepsy.

Here, we identified *Fgf9* as a new candidate gene responsible for epileptogenesis. *Fgf9* deletion in GABAergic neurons caused neuronal apoptosis and decreased the expression of GABA by activating the adenylatecyclase (AC)/ cyclic AMP (cAMP) and extracellular signal-regulated kinase (ERK) signaling pathway, causing a GABA/Glu imbalance and inducing epilepsy in mice. To our knowledge, this is the first study to establish a clear causal linkage between *Fgf9* loss-of-function and epilepsy. In addition, the decrease of FGF9 in serum was commonly observed in human epileptic patients, especially with focal seizure. Those findings provide a new explanation of epilepsy and pave the way for the design of novel therapeutics for epilepsy.

## Results

### *Olig1*-cre mice with conditional knockout (CKO) of *Fgf9* exhibited epilepsy

**Fig. 1:**
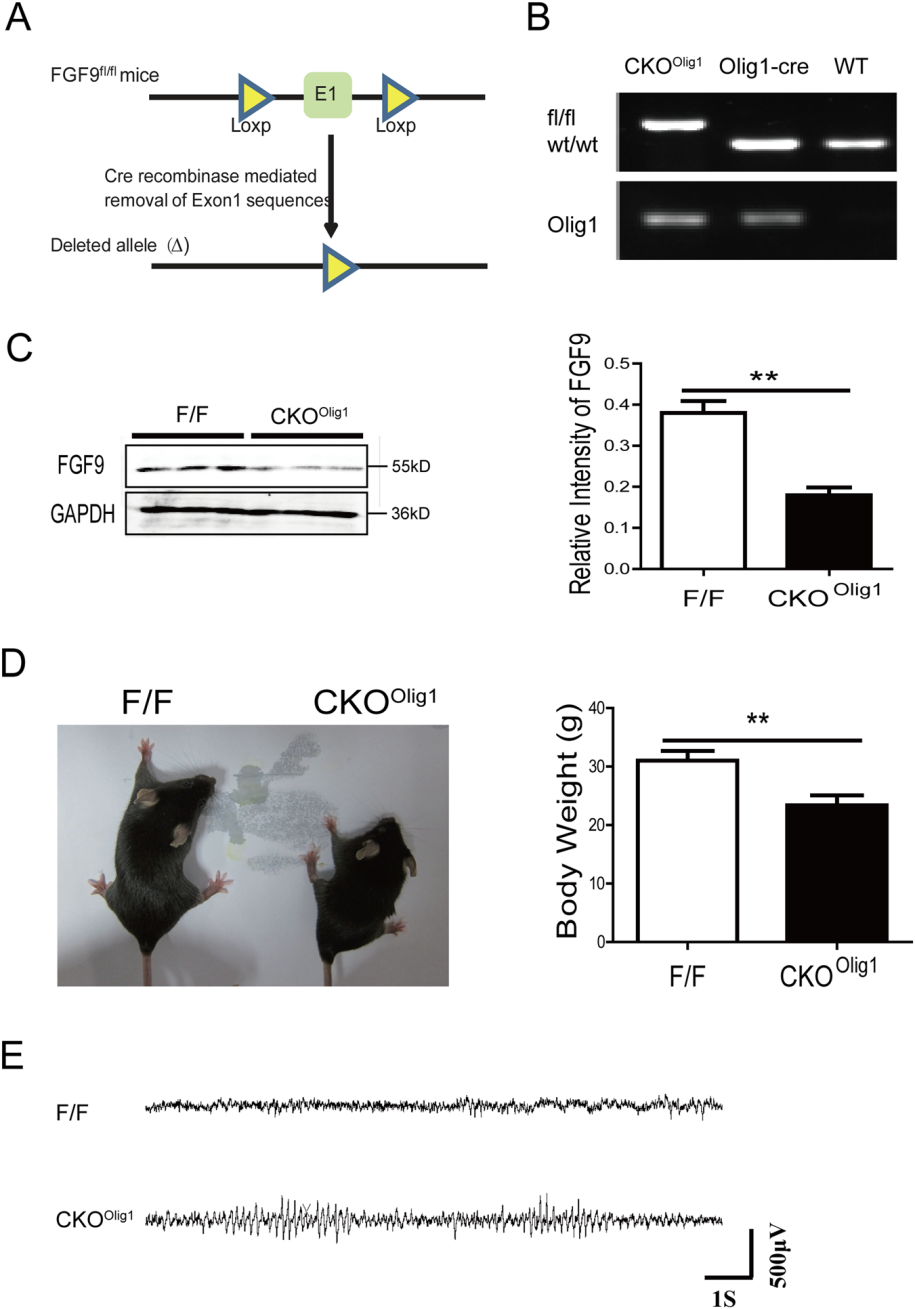
*Fgf9 CKO* in *Olig1*-Cre mice causes epilepsy. **A** Schematic diagram describes the design of the floxed and *CKO Fgf9* allele. Coding exons are shown in green boxes. **B** PCR analyses show the genotyping in the *Fgf9*^*loxP/loxP*^*Olig1*-cre (*CKO*^*Olig1*^), *Olig1-Cre*, and wild-type mice. **C** Western blot analyses demonstrate a decrease in FGF9 expression in the *CKO*^*Olig1*^ brain compared to that in *Fgf9*^*flox/flox*^ (*F/F*) controls at 8 weeks of age. GAPDH was used as the internal loading control. **D** *CKO*^*Olig1*^ mice exhibited growth retardation at 8 weeks of age. Data are mean ± SEM for 10 mice per genotype. *P < 0.05; **P < 0.01, nonparametric Mann-Whitney test. **E** EEG recordings from *CKO*^*Olig1*^ mice showing spontaneous seizures. Data represent mean ± SEM of triplicate samples.

*Fgf9* homozygous knockout leads to neonatal death of *Fgf9*-null mice due to lung failure^11^. To study the effects of FGF9 on epileptogenesis, we generated *Fgf9 CKO* mice in oligodendrocyte progenitor cells that express *Oligo1* (*Fgf9*^*fl/fl*^*Olig1-Cre*, *CKO*^*Olig1*^) (Fig. 1A), and verified by genotyping and examining protein expression in the brain (Fig. 1B, C). The expression of FGF9 was dramatically lower in the *CKO*^*Olig1*^ brains (mean ± SEM, 0.379 ± 0.029) than in the *Fgf9*^*fl/fl*^ (*F/F*) brains (0.179 ± 0.019, *P* = 0.0133, *n* = 3).

We observed severe spontaneous seizures as early as 2–3 weeks after birth in *CKO*^*Olig1*^ mice (Video 1). Strikingly, the spontaneously arising seizures showed a pattern of repetitive low amplitude spike-wave (4–7 Hz) on electroencephalograms (EEGs) (Fig. 1E). We also carried out a series of behavioral measurements in *CKO*^*Olig1*^ mice. Their behavioral abnormalities included: large clonus of all limbs; dystonic or hypertonic posture of the trunk, limbs and tail, often asymmetric; and four-limb tonic-clonic hypertonic postures. Interestingly, all *CKO*^*Olig1*^ mice were smaller than littermate controls (23.45 ± 1.54 g vs 31.75 ± 1.40 g, *P* = 0.004, *n* = 10) but had a normal lifespan (Fig. 1D). In contrast, heterozygous *Fgf9 CKO* mice and *F/F* littermates never showed epileptic seizures or behavioral abnormalities. *F/F* mice were used as the control in this study. Together, these results indicate that *CKO*^*Olig1*^ mice exhibit severe epileptic seizures.

### Abnormal oligodendrocytes were found in the cortex of *CKO*^*Olig1*^ mice

**Fig. 2:**
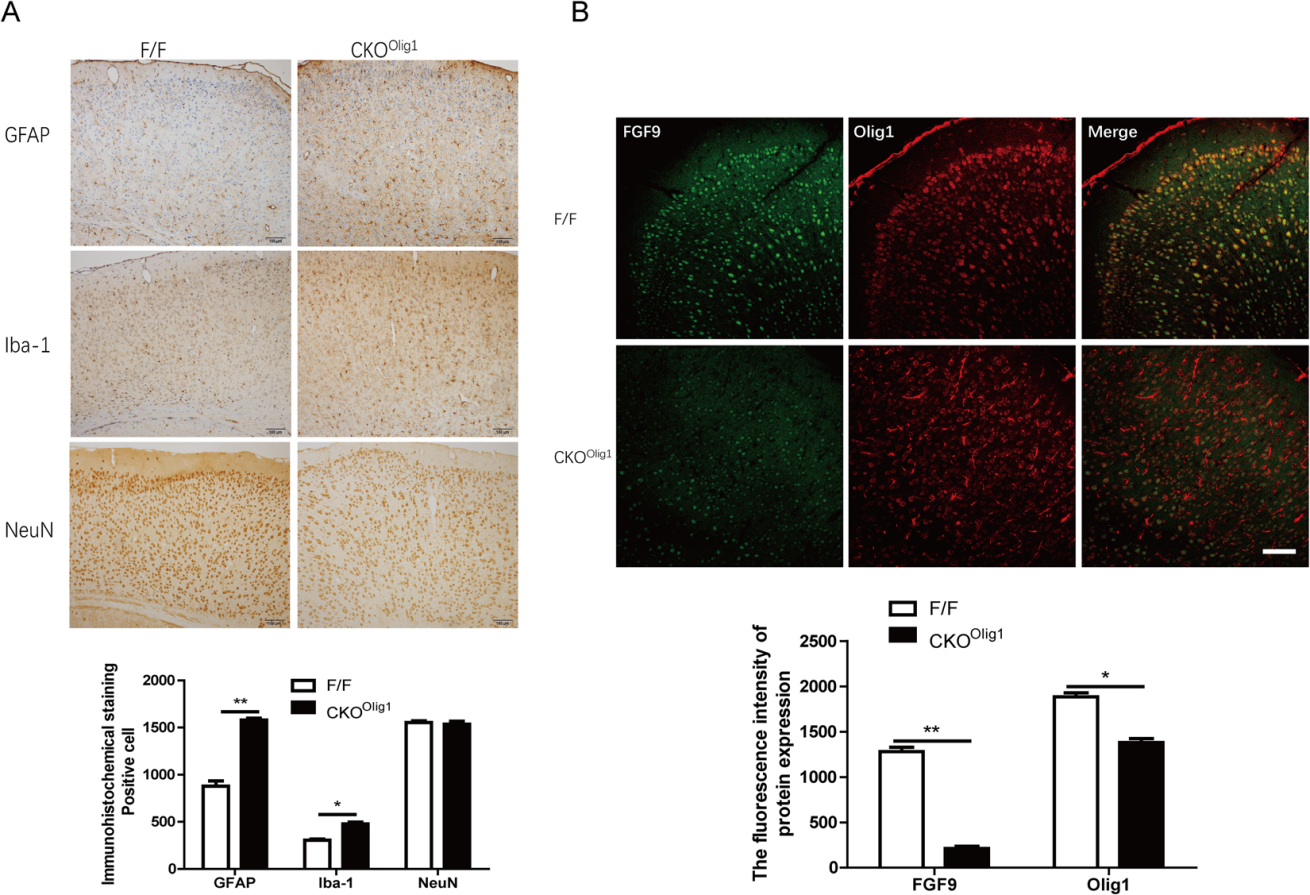
Abnormal pathological changes are found in the cortex of *CKO*^*Olig1*^ mice. **A** Immunohistochemical staining for astrocytes, microglia and neurons in the cortex of *CKO*^*Olig1*^ mice and *F/F* mice. The brain area in the figure is the motor cortex. **B** Immunostaining for FGF9 and Olig1 in the cortex of *CKO*^*Olig1*^ mice. The density of FGF9 (1281 ± 47.4 vs 211 ± 25.7, *P* = 0.0018) or Olig1 (1885 ± 43.7 vs 1380 ± 43.9, *P* = 0.0287) in the cortex of *F/F* and *CKO*^*Olig1*^ mice. Scale bars, 100 μm.

Pathological changes in the cortex, hippocampus, and thalamus are considered to play important roles in epileptic seizures; therefore, we examined the expression patterns of NeuN, Iba-1, and GFAP, as markers of neuron, microglia, and astrocytes, respectively, in CNS of *CKO*^*Olig1*^ mice. Compared to *F/F* mice, *CKO*^*Olig1*^ mice exhibited obviously inflammatory pathological changes including increased number of astrocytes (1560 ± 23 vs 884 ± 32, *P* = 0.0052, *n* = 3) and microglia (454 ± 14 vs 320 ± 16, *P* = 0.0144) in the cortex (Fig. 2A), but the number of neurons (1553 ± 25 vs 1566 ± 16, *n* = 3) were not obviously altered. Immunohistochemical staining did not reveal any abnormalities to be observed in the thalamus or hippocampus of *CKO*^*Olig1*^ and *F/F* mice (Fig. S2). To identify alterations in the cortex of *CKO*^*Olig1*^ mice, we performed FGF9 and Olig1 double immunostaining. The cortex of *CKO*^*Olig1*^ mice had significantly fewer FGF9-positive cells than that of *F/F* mice, and FGF9 labeling was not localized with Olig1. Interestingly, abnormal Olig1-positive cell morphology was displayed in the cortex of *CKO*^*Olig1*^ mice (Fig. 2B). Hence, the epilepsy in *CKO*^*Olig1*^ mice is related to pathological changes in the cortex, especially abnormal Olig1-positive cells.

### *Fgf9 CKO* in GABAergic neurons or their progenitor cells led to seizures

Olig1 regulates the differentiation and myelination of oligodendrocytes, as well as the commitment of early oligodendrocyte progenitor cells^12^. We first hypothesized that the epilepsy in *CKO*^*Olig1*^ mice might be caused by demyelination due to oligodendrocyte dysfunction. To address this, we examined demyelination in the brain and spinal cord of *CKO*^*Olig1*^ mice by Luxol fast blue (LFB). Surprisingly, the staining degree of LFB did not show significant demyelination in the hippocampus or spinal cord of *CKO*^*Olig1*^ mice and *F/F* mice (Fig. S1). Therefore, the spontaneous seizures of the *CKO*^*Olig1*^ mice were not a result of demyelination.

**Fig. 3:**
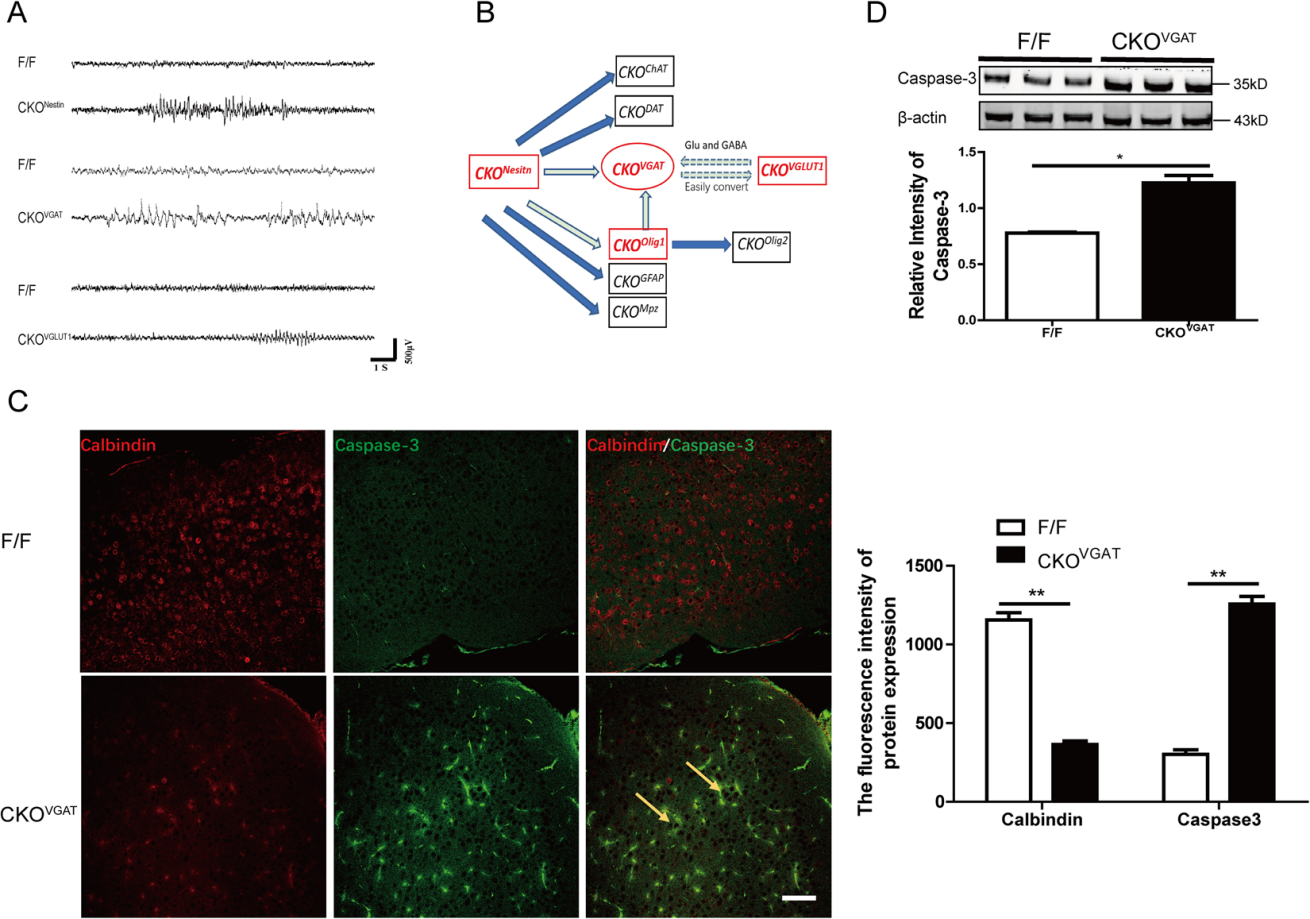
*Fgf9 CKO* in GABAergic neurons induces neuronal apoptosis. **A** EEG recordings of other types of *Fgf9 CKO* mice associated with GABAergic neurons (*CKO*^*Nestin*^, *CKO*^*VGAT*^, and *CKO*^*VGLUT1*^) showing spontaneous seizures. **B** Schematic diagram describes that *CKO Fgf9* in many different cell types. *CKO* mice with epilepsy indicated in red, and no seizure *CKO* mice indicated in black. **C** Caspase-3 colocalized with GABAergic neurons (calbindin) in the cortex of *CKO*^*VGAT*^ mice by immunostaining. The density of calbindin (1156 ± 45.6 vs 364 ± 22.3, *P* = 0.0054) or caspase-3 (302 ± 28.1 vs 1324 ± 25.2, *P* = 0.0063) in the cortex of *F/F* and *CKO*^*VGAT*^ mice. Scale bars, 100 μm. **D** Western blot analysis demonstrates higher caspase-3 expression in the cortex of *CKO*^*VGAT*^ mice than in controls.

The progenitor cells within the ventral telencephalon (expressing Olig1) can generate GABAergic neurons and oligodendrocytes. To determine whether epilepsy in *CKO*^*Olig1*^ mice is associated with GABAergic neurons, we generated a series of conditional *Fgf9* knockout mice. *Fgf*9 was specifically knocked-out in GABAergic neurons in *CKO*^*VGAT*^ mice, neural progenitor cells in *CKO*^*Nestin*^ mice, excitatory glutamatergic neurons in *CKO*^*VGLUT1*^ mice, astrocytes in *CKO*^*GFAP*^ mice, motor neurons and oligodendrocytes in *CKO*^*Olig2*^ mice, dopaminergic neurons in *CKO*^*DAT*^ mice, Schwann cell in *CKO*^*Mpz*^ mice, and cholinergic neurons in *CKO*^*ChAT*^ mice. Among those, *CKO*^*Nestin*^, *CKO*^*VGAT*^, and *CKO*^*VGLUT1*^ mice exhibited spontaneous seizures (Fig. 3A, B, Table 1, and Videos 2–4); other *CKO* mice were no different from their littermates.

**Table 1:** Summary of seizure and EEG and characteristics of seizures in multiple *CKO* mice.

Genotype	Number of mice with seizures (total *n*)		Mice with seizures (%)	Age range at first seizure (days)	Frequency range of seizures (/d)^a^	Mortality (%)
	EEG	Seizures				
*CKO*^*Olig1*^	3 (3)	17 (17)	100	21–36	4–18	0
*CKO*^*VGAT*^	3 (3)	18 (18)	100	16–30	9–26	44
*CKO*^*Nestin*^	3 (3)	11 (11)	100	20–38	3–12	0
*CKO*^*VGLUT1*^	3 (3)	12 (12)	100	22–28	2–5	0
*CKO*^*Chat*^	0 (3)	0 (12)	0	NA	NA	0
*CKO*^*Olig2*^	0 (3)	0 (12)	0	NA	NA	0
*CKO*^*GFAP*^	0 (3)	0 (11)	0	NA	NA	0
*CKO*^*DAT*^	0 (3)	0 (11)	0	NA	NA	0
*CKO*^*Mpz*^	0 (3)	0 (11)	0	NA	NA	0
*F/F* (controls)	0 (3)	0 (18)	0	NA	NA	0

^a^Data from 3–4 week old *CKO* mice.

Among the epileptic mice, the *CKO*^*VGAT*^ mice had the highest frequency of seizures and the most severe form of epilepsy, with a 1-month mortality rate of 44% (Table 1). All the behavioral changes in *CKO*^*VGAT*^ mice were typically accompanied by EEG abnormalities (Video 5). EEG recordings of *F/F* mice and *CKO*^*VGAT*^ mice during the incubation period did no differ significantly at baseline (Videos 6 and 7). Both *CKO*^*Nestin*^ and *CKO*^*VGAT*^ mice were smaller than their littermate controls, in particular *CKO*^*VGAT*^ mice lost nearly half their weight (Fig. S3A, B). Interestingly, the epileptic seizures of *CKO*^*VGLUT1*^ mice were mild and transient and only occurred between 3 and 4 weeks of age. Taken together, these observations show that *Fgf9* depletion in GABAergic neurons or their progenitor cells (Nestin or Olig1) leads to severe spontaneous seizures in mice, suggesting a causal relationship between *Fgf9* knockout and abnormal GABAergic neurons.

### *Fgf9 CKO* in GABAergic neurons induced apoptosis and caused a GABA/Glu imbalance in the cortex

To determine whether *FGF9* ablation in GABAergic neurons induces apoptosis, we assessed calbindin (immunoreactive GABAergic neuron marker) and caspase-3 (marker of apoptosis activation) expression by double immunostaining in the cortex. Compared to *F/F* mice, *CKO*^*VGAT*^ mice had significantly fewer GABAergic neurons in the cortex, the fluorescence intensity of calbindin decreased approximately 70%, and notably more GABAergic neurons coexpressing caspase-3 (Fig. 3C). Meanwhile, a marked increase in caspase-3 expression was detected at the protein level in *CKO*^*VGAT*^ mice than that in *F/F* mice (1.225 ± 0.067 vs 0.776 ± 0.010, *P* = 0.0276, *n* = 3) (Fig. 3D). These results show that FGF9 plays a crucial role in the survival of GABAergic neurons.

**Fig. 4:**
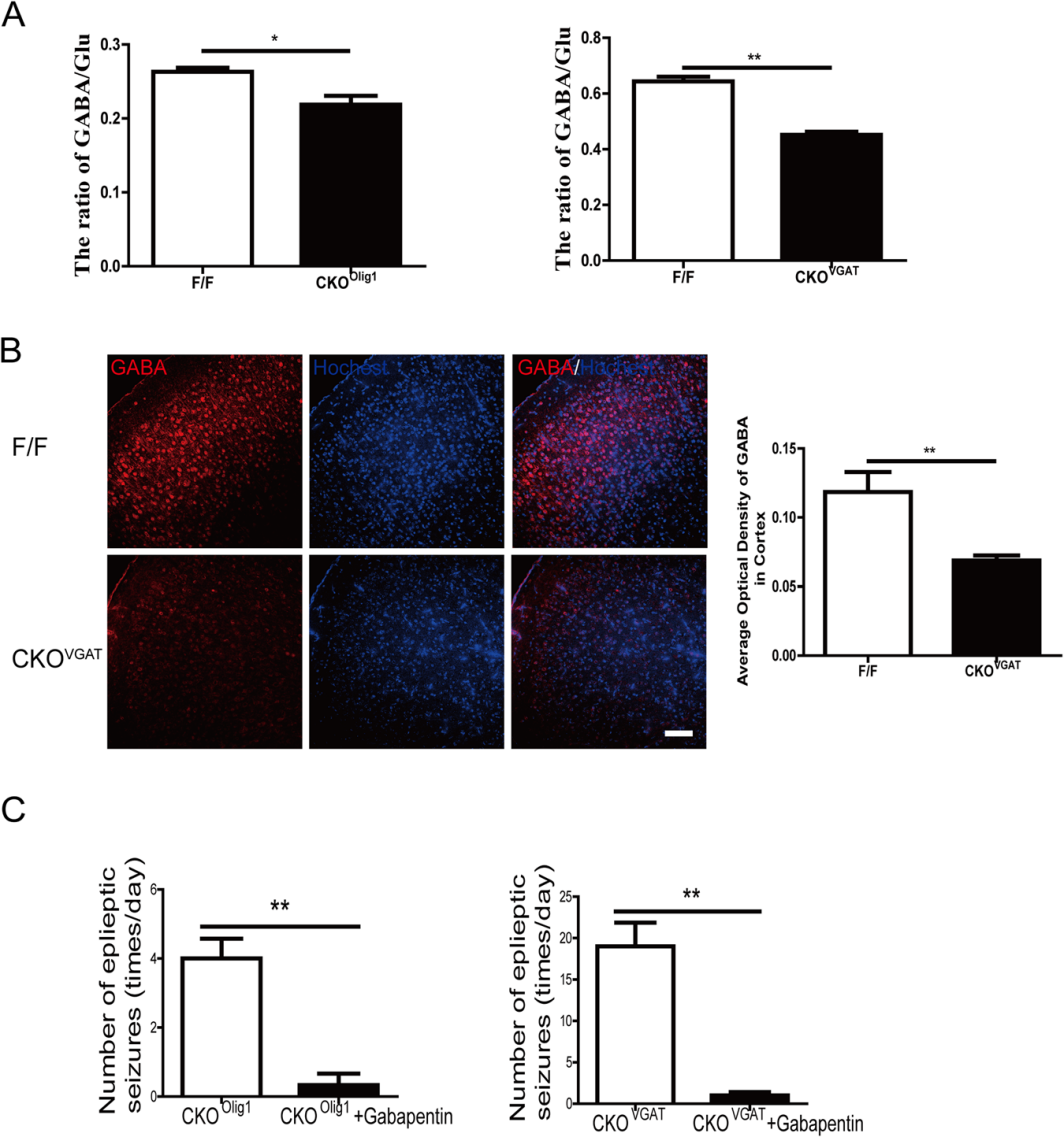
*Fgf*9 CKO in GABAergic neurons decreases GABA release. **A** The ratio of GABA/Glu in the cortex was lower in *CKO*^*Olig1*^ and *CKO*^*VGAT*^ mice than in control mice. Data are mean ± SEM for 3 mice per genotype. *P < 0.05; **P < 0.01, nonparametric Mann-Whitney test. **B** Confocal images of GABA in the cortex of *CKO*^*VGAT*^ mice. Scale bars, 100 μm. **C** Administration of gabapentin significantly reduced the frequency of epileptic seizures in *CKO*^*Olig1*^ (*n* = 3) and *CKO*^*VGAT*^ mice (*n* = 4). Significant differences from the control: *P < 0.05; **P < 0.01, paired Student’s *t*-test.

Next, we measured the content of GABA and Glu in the cortex of *CKO*^*Olig1*^ and *CKO*^*VGAT*^ mice with the aid of ultra-high-performance liquid chromatography tandem mass spectrometry (UHPLC-MS/MS). The Glu concentration was similar in the cortex of the *CKO* mice and control mice, but the GABA concentration was lower in *CKO* mice, especially *CKO*^*VGAT*^ mice, than in the control mice (Fig. S4A, B). The GABA/Glu ratios in the cortex of *CKO*^*Olig1*^ and *CKO*^*VGAT*^ mice were significantly lower than those in the controls (0.22 ± 0.012 vs 0.26 ± 0.005, *P* = 0.0198, and 0.45 ± 0.012 vs 0.64 ± 0.016, *P* = 0.0051, *n* = 3) (Fig. 4A). The immunofluorescence results also showed that GABA expression was significantly lower in the cortex of *CKO*^*VGAT*^ mice than in that of control mice (Fig. 4B). In brief, these results indicate that *Fgf9 CKO* reduces the expression of GABA in the cortex, resulting in an imbalance of GABA and Glu.

To further characterize the reduction in GABA-mediated epileptogenesis, we gave *Fgf9 CKO* mice an intraperitoneal injection of gabapentin, leading to an overall increase in GABA levels in the CNS. Both *CKO*^*Olig1*^ and *CKO*^*VGAT*^ mice became almost seizure-free after gabapentin administration (Fig. 4C). The antiepileptic effect began to appear half an hour after gabapentin administration and lasted for about 12 h. Thus, epilepsy in *Fgf9 CKO* mice is closely related to the imbalance in GABA/Glu, potentially caused by diminished concentration of GABA.

### The AC/cAMP signaling pathway involved in epilepsy in *CKO* mice

**Fig. 5:**
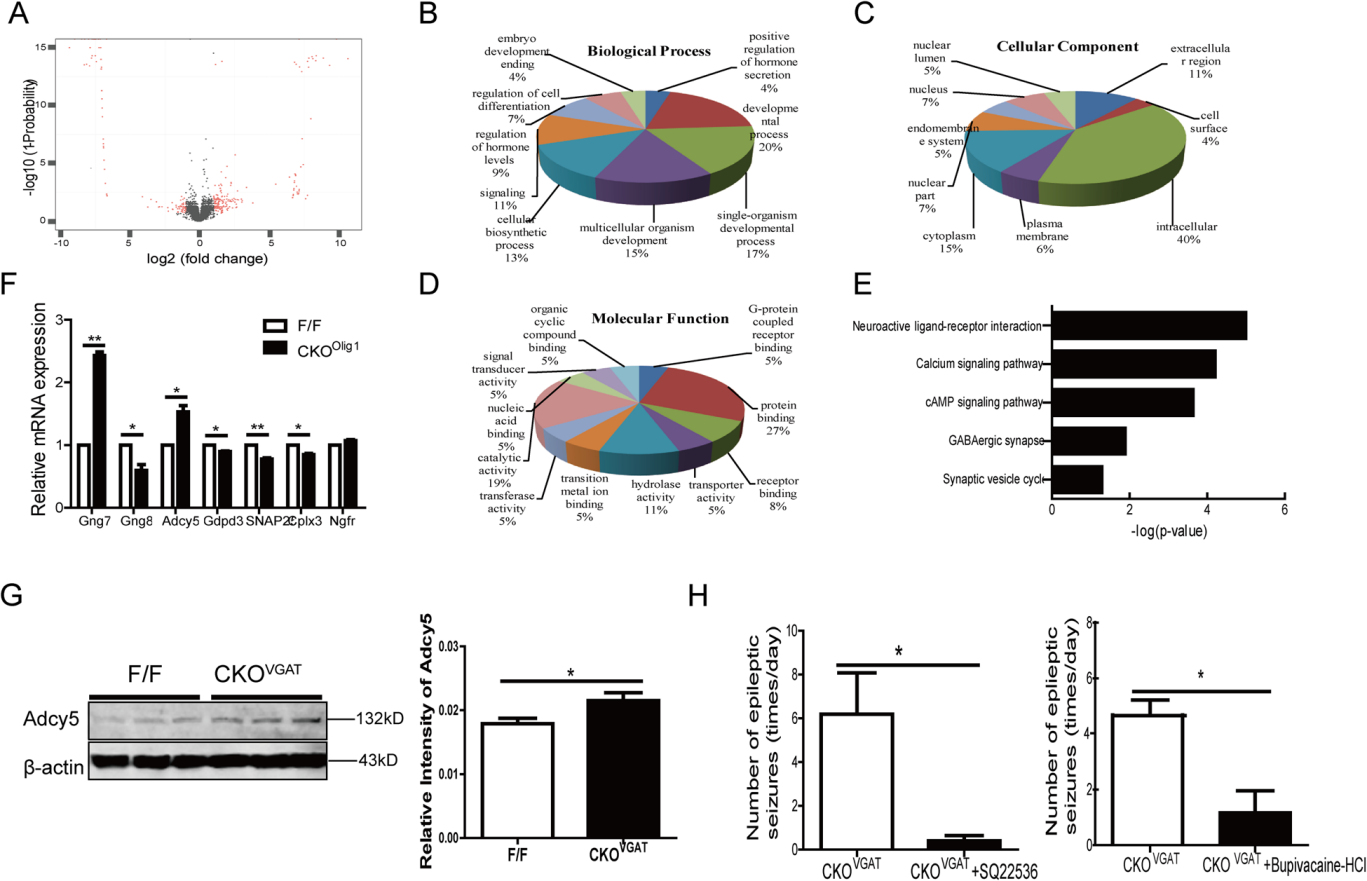
RNA-seq analysis of the DEGs and signaling pathways between *CKO*^*Olig1*^ and *F/F* mice. **A** Volcano plot to visually compare the DEGs between the *CKO*^*Olig1*^ group and the control group. **B–D** GO analysis of the cellular components, molecular functions and biological processes associated with the DEGs. **E** KEGG pathways of DEGs between the *CKO*^*Olig1*^ and *F/F* mice. **F** qRT-PCR validation of some DEGs. **G** Western blot analyses demonstrate higher in Adcy5 expression in *CKO*^*VGAT*^ mice than in controls. β-actin was used as the internal loading control. Data are mean ± SEM for 3 mice per genotype. **H** The SQ22536/bupivacaine-HCl (AC/cAMP inhibitor) significantly reduced the frequency of seizures in *CKO*^*VGAT*^ mice (*n* = 5 and *n* = 6). Significant differences from the control group: *P < 0.05; **P < 0.01, paired Student’s *t*-test.

To further investigate the molecular mechanism of *Fgf9 CKO*-induced epilepsy, we compared the gene expression profiles in the cortex of *CKO*^*Olig1*^ and control mice by RNA sequencing (RNA-seq). RNA-seq data revealed significant changes in gene expression: 223 (65.6%) transcripts were upregulated, and 117 (34.4%) transcripts were downregulated in *CKO*^*Olig1*^ mice (Fig. 5A, Fig. S5B, and Tables S1 and S2). Approximately 16,600 genes were commonly expressed between the *CKO* and control mice (Fig. S5A). We evaluated three Gene Ontology (GO) categories: biological process, cell component, and molecular function. The differentially expressed genes (DEGs) were mostly involved in biological processes such as the developmental process, cellular biosynthetic process, signaling pathway, regulation of hormone levels, and regulation of cell differentiation (Fig. 5B). In terms of cellular components, the DEGs were involved in the intracellular region, extracellular region, and cytoplasm (Fig. 5C). The molecular functions identified by the DEGs were protein binding, catalytic activity, and hydrolase activity (Fig. 5D). Kyoto Encyclopedia of Genes and Genome (KEGG) pathway enrichment analysis showed that the DEGs were involved in neuroactive ligand–receptor interaction, the calcium signaling pathway, the cAMP signaling pathway, and GABAergic synapse (Fig. 5E). Most notably, DEGs involved in AC (Adcy)/cAMP signaling pathway, such as *Gng7* and *Adcy5*, were significantly upregulated in *CKO*^*Olig1*^ mice (Fig. S5C). To confirm the RNA-seq results, we selected seven DEGs for validation by qRT-PCR, including *Gng7*, *Gng8*, *Adcy5*, *Snap25*, *Cplx3*, *Cdpd3*, and *Ngfr*. We found similar trends and fold changes for most genes by real-time PCR (qPCR) and RNA-seq (Fig. 5F). In particular, we also detected a marked increase in Adcy5 expression at the protein level in *CKO*^*VGAT*^ mice (Fig. 5G).

Given that AC/cAMP signaling is activated in *CKO*^*Olig1*^ and *CKO*^*VGAT*^ mice, we asked whether inhibiting the AC/cAMP pathway could yield therapeutic benefit. We treated *CKO*^*VGAT*^ mice with SQ22536 or bupivacaine-HCl, an inhibitor of AC or cAMP production, to reduce the level of cAMP in the tissue. Both SQ22536 and bupivacaine-HCl significantly reduced the frequency of seizures (Fig. 5H). These results suggest that inhibition of the AC/cAMP signaling pathway can suppress seizures in *CKO*^*Olig1*^ and *CKO*^*VGAT*^ mice.

**Fig. 6:**
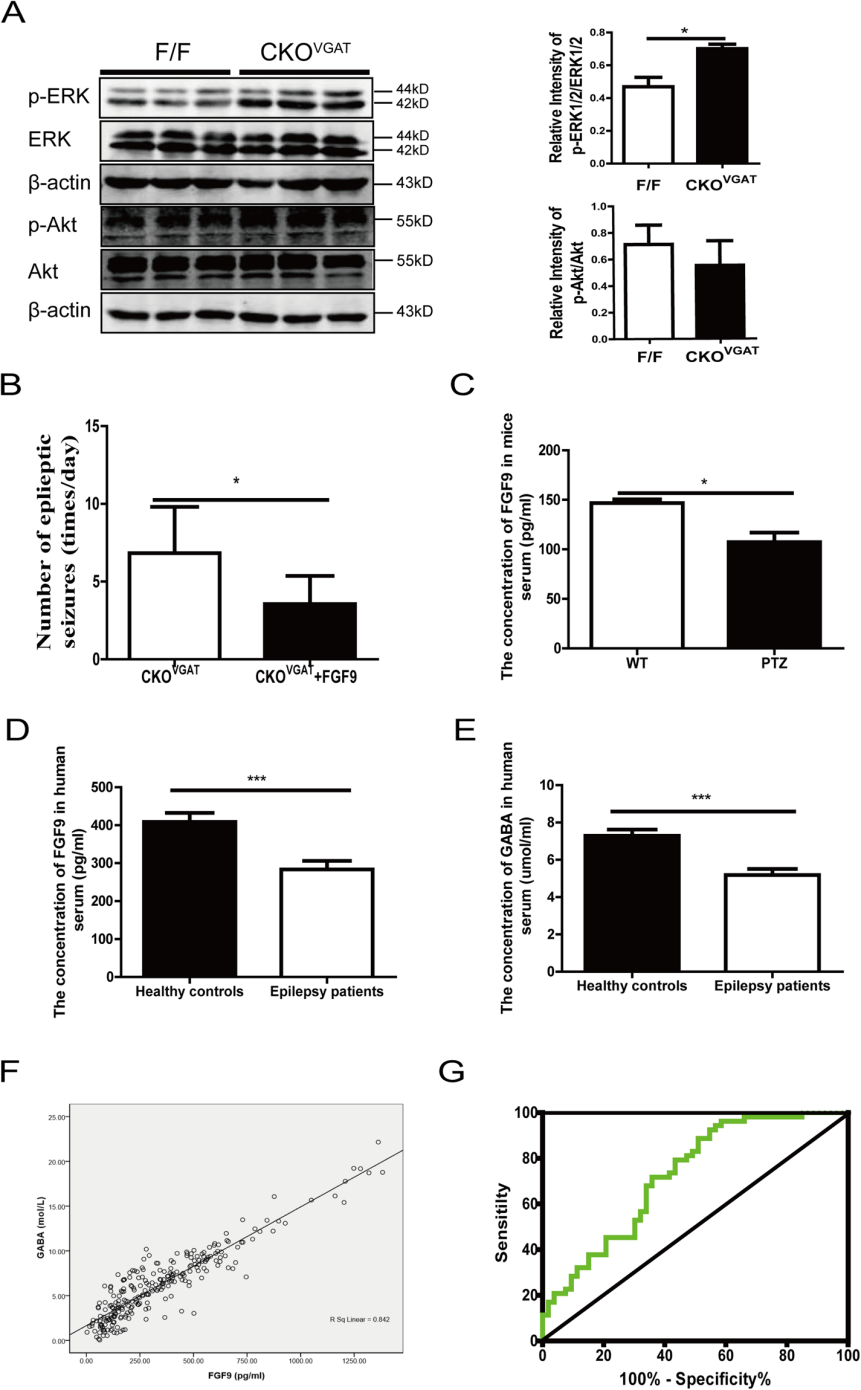
Decreased expression of FGF9 is a common mechanism in epilepsy. **A** The ERK1/2 pathway was activated in *CKO*^*VGAT*^ mice. Each test was repeated 3 times. β-actin was used as the internal loading control. Data are presented as mean ± SEM; *P < 0.05 and **P < 0.01. **B** Administration of rhFGF9 proteoliposomes significantly reduced the frequency of seizures in *CKO*^*VGAT*^ mice (*n* = 6). Significant differences from the control: *P < 0.05, paired Student’s *t*-test. **C** The expression of FGF9 determined by ELISA was lower in the serum of PTZ-induced mice (*n* = 5) that in that of control mice. **D**, **E** ELISA to quantify FGF9 and GABA levels in serum from 128 controls and 128 epileptic patients. **F** The correlation between the serum expression of FGF9 and GABA was significant (*r*^2^ = 0.842). **G** ROC curve analysis showed the diagnostic significance of FGF9 in patients with focal seizures (AUC = 0.725).

The extracellular signal-regulated kinase 1/2 (ERK1/2) and Akt signaling pathways are involved in cell survival proliferation and apoptosis; therefore, we analyzed the phosphorylation statuses of ERK1/2 and Akt. Phosphorylated (p-) ERK1/2 expression was higher in the cortex of *CKO*^*VGAT*^ mice than in that of control mice, but no change in p-Akt expression was shown by western blot (Fig. 6A). These results suggest that the ERK1/2 signaling pathway plays an important role in GABAergic neuron apoptosis.

### Protective effect of FGF9 on the seizures of *CKO*^*VGAT*^ mice

To confirm the protective effect of FGF9, recombinant human FGF9 (rhFGF9) proteoliposomes were intranasally administered immediately in *CKO*^*VGAT*^ mice. The *CKO*^*VGAT*^ mice were videotaped, and the number of seizures was counted about 72 h before or after administration. Compared to the previous untreated *CKO*^*VGAT*^ mice, the rhFGF9 proteoliposomes-treated *CKO*^*VGAT*^ mice had significantly fewer daily seizure counts (Fig. 6B). These results suggest that FGF9 is expected to serve as a new target for epilepsy treatment.

### Changes of FGF9 were related to epilepsy

To explore the involvement of FGF9 in epilepsy, we used a pentylenetetrazole (PTZ)-kindled mouse epileptic model and epileptic patients. PTZ-kindled mice expressed lower levels of FGF9 in the serum (by ELISA kit) and the cortex (by western blot) than control mice (Fig. 6C and Fig. S6A). Furthermore, PTZ-kindled mice also elevated Adcy5 protein levels in the cortex (Fig. S6A).

The protein expression levels of serum FGF9 and GABA in epileptic patients (*n* = 128) and healthy controls (*n* = 128) matched by age and gender were detected by ELISA (elsbiotech, China). As shown in Fig. 6D and Table 2, the expression of serum FGF9 in epileptic patients was significantly lower than that in healthy controls (283.7 ± 254.8 vs 408.4 ± 268.3, *P* < 0.001), as was the expression of serum GABA (5.19 ± 3.71 vs 7.29 ± 3.78, *P* < 0.001) (Fig. 6E). Serum FGF9 and GABA levels were significantly correlated in epileptic patients and controls (Fig. 6F). Additionally, the mRNA and protein expression levels of FGF9 in 21 epileptic patients and controls were basically consistent with the ELISA results (71.4% and 85.7%) (Fig. S6B, C).

**Table 2:** The association between the expression of serum FGF9, GABA, and epileptic seizures.

	Variables	Control cases (*n* = 128)	Epileptic patients (*n* = 128)	*P* value
Information	Mean age (year)	38.1 ± 18.7	36.6 ± 19.2	
	Male, *n* (%)	71 (55.4%)	71 (55.4%)	
	Mean weight (kg)	66 ± 14.4	66.8 ± 18.2	0.5376
	Mean height (cm)	166 ± 11	165 ± 14.3	0.9020
Content	FGF9 in serum (pg/mL)	408.4 ± 268.3	283.7 ± 254.8	<0.001
	GABA in serum (μmol/L)	7.29 ± 3.78	5.19 ± 3.71	<0.001
Epilepsy types	Focal		53	<0.0001
	Combined generalized and focal		13	0.0138
	Generalized		7	0.3176
	Unknown		55	0.0185

The epileptic seizures were classified according to the International League Against Epilepsy Classification^13^. Seizures were registered in 128 patients (focal seizures: 53 cases (41.41%), combined generalized and focal seizures: 13 cases (10.16%), generalized seizures: 7 cases (5.47%), and unknown group: 55 cases (42.97%)). The results in Table 2 show that FGF9 expression is significantly lower in those with focal seizures (*P* value < 0.0001), combined generalized and focal seizures (*P* value = 0.0138) and unknown seizures (*P* value = 0.0185). A receiver operating characteristic (ROC) curve with FGF9 was built to investigate the diagnostic value of serum FGF9 in focal seizures. The area under the curve (AUC) of FGF9 was 0.725, which revealed that the diagnostic value of FGF9 was effective for those with focal seizures (Fig. 6G).

## Discussion

FGFs are involved in many biological activities, including organ development, cell growth, morphogenesis, tissue repair, tumor growth, and metabolism^14–16^. FGF2, FGF5, FGF8, and FGF9 are widely expressed in the CNS, and play important roles in neurogenesis, differentiation, and survival^17^. However, very little is known about the impact of FGF9 on epileptogenesis. In our study, we found that mice with *Fgf9 CKO* in oligodendrocyte progenitor cells (Olig1) developed seizures (Fig. 1), and pathological changes in the cortex, including abnormal Olig1-positive cells and microglial and astrocytic activation (Fig. 2).

Olig1 and olig2 have been identified as essential factors in the specification of oligodendroglia^18^. Olig2 is a master regulator for oligodendrocyte (OL) lineage and some neurons’ development^12^. The role of Olig1 in the specification phase of oligodendrocyte formation is partially redundant in the presence of Olig2, because ablation of Olig1 has no impact on the formation of motor neurons and early oligodendrocyte progenitors^19^. In our study, no evidence of demyelination in spinal cords of *CKO*^*Olig1*^ mice was observed (Fig. S1), probably due to the partial functional compensation by Olig2. These results are consistent with the cell-intrinsic activity of Olig1, and can be compensated for during myelination^20^.

A series of *CKO* mice (*CKO*^*VGAT*^ mice, *CKO*^*Nestin*^ mice, *CKO*^*VGLUT1*^ mice, *CKO*^*GFAP*^ mice, *CKO*^*Olig2*^ mice, *CKO*^DAT^ mice, *CKO*^*Mpz*^ mice, and *CKO*^*Chat*^ mice) were generated to determine what type of cells knockout FGF9 is associated with the epilepsy in *CKO*^*Olig1*^ mice. Among those, *CKO*^*Nestin*^, *CKO*^*VGAT*^, and *CKO*^*VGLUT1*^ mice exhibited spontaneous seizures, whereas other *CKO* mice were not different from their littermates (Fig. 3 and Table 1). Previous studies have revealed that Olig1-positive progenitors could generate GABAergic neurons and oligodendrocytes^21^. In our study, epilepsy was found in FGF9 knockout in Olig1-postive progenitors rather than in Olig2-postive progenitors, which regulates the sequential specification of neural progenitor cells into motor neurons and oligodendrocyte precursor cells^22^. Nestin marks neural stem cells, which can generate neurons (including GABAergic neurons) and glial cells^23,24^. The vesicular GABA transporter (VGAT) is an essential molecule for GABAergic neurotransmission due to its role in vesicular GABA release^25,26^. Therefore, the *Fgf9* depletion in all *CKO* mice with seizures was related to GABAergic neurons or their progenitor cells (Nestin or Olig1-postive cells). Furthermore, the *CKO*^*VGAT*^ mice exhibited the most severe growth retardation and seizures, even leading to death. VGLUT1 is often used for studying glutamatergic synaptic vesicle trafficking in glutamatergic neurons^27^. On the other hand, the biochemical relationship between Glu and GABA goes far beyond this reciprocally modulating activity, since inhibitory presynaptic neurons easily convert Glu to GABA through glutamic acid decarboxylase (GAD)^28^.We anticipated that the synthesis of GABA from Glu might be impaired in *CKO*^*VGLUT1*^ mice; however, the interference may be partially compensated^28^. Future research on epilepsy in *CKO*^*VGLUT1*^mice may be interesting.

We discovered reduced expression of GABA in *CKO*^*VGAT*^ mice by UHPLC-MS/MS and immunofluorescence (Figs. S4A and S4B). Epilepsy in *Fgf9 CKO* mice is closely related to GABA/Glu imbalance, potentially caused by a decrease in the concentration of GABA^29^. Gabapentin was initially synthesized to mimic the chemical structure of GABA^30^, but it neither binds to GABAA or GABAB receptors nor is metabolized into GABA^31^. The effects of gabapentin on GABA synthetic and metabolizing enzymes reveal a complex pattern of activity, potentially, leading to an overall increase in central GABA levels^32^. In our study, gabapentin administration significantly reduced the frequency of seizures in both *CKO*^*Olig1*^ and *CKO*^*VGAT*^ mice (Fig. 4C), supporting the notion that gabapentin elevates GABA in the CNS and offers protection against further seizures in patients^33^.

GABAergic neurons can release GABA to bind a receptor on excitatory neurons that restrains “firing”, and impaired GABAergic function has been established as a cause of epilepsy^34^. In our research, caspase-3 expression was found to be elevated in GABAergic neurons of *CKO*^*VGAT*^ mice (Fig. 3C, D), supporting the notion that GABAergic neuron loss and triggering glia-mediated neuroinflammation to increase neuronal death in the cortex of *CKO*^*VGAT*^ mice, consistent with our previous finding that FGF9 played a key role in the survival and development of Purkinje cells in cerebellum^35^. In addition, rhFGF9 proteoliposomes administration significantly reduced the frequency of seizures in *CKO*^*VGAT*^ mice (Fig. 6B), confirming that FGF9 may have antiepileptic effects.

RNA-seq is a practical technique for high-throughput gene expression and function studies^36^. In our study, a total of 340 DEGs were detected in the cortex (223 upregulated and 117 downregulated) between *F/F* and *CKO*^*Olig1*^ mice. KEGG pathway analysis showed that the DEGs involved in AC/cAMP signaling pathway was significantly increased in *CKO*^*Olig1*^ mice (Fig. 5). As a second messenger, cAMP ubiquitously regulates many kinds of cellular processes. The elevation of cAMP has dual roles, resulting in proliferative and proapoptotic effects^37,38^. We treated *CKO*^*VGAT*^ mice with SQ22536 or bupivacaine-HCl, the inhibitor of AC or cAMP. Here, SQ22536 and bupivacaine-HCl effectively reduces seizures in *CKO*^*VGAT*^ mice (Fig. 5H), consistent with the previous finding that activation of AC/cAMP signaling promoted epileptic seizures in mice and humans^39–42^. In addition, cross-talk between cAMP and ERK has been demonstrated in many studies. For instance, cAMP can inhibit ERK or activate ERK in a cell-specific manner^43^. In our results, p-ERK1/2 were activated in the cortex of *CKO*^*VGAT*^ mice (Fig. 6A), suggesting that AC/cAMP/ERK pathway is involved in the apoptosis of GABAergic neurons.

In the present study, we detected the expression of FGF9 in the serum in PTZ-kindled seizure model mice and epileptic patients. FGF9 expression was significantly lower in the serum and cortex of PTZ-kindled mice than in those of control mice, and Adcy5 expression was higher (Fig. S6A). Furthermore, we detected the expression of serum FGF9, revealing that it was significantly lower in epileptic patients than that in healthy controls (Fig. 6D). These observations suggested that FGF9 was universally implicated in epileptogenesis. Meanwhile, there was a good correlation between FGF9 and GABA in the serum (Fig. 6E, F), which strengthened the idea that *Fgf9* loss-of-function causes an imbalance of GABA and Glu and induces epilepsy. These findings strongly suggest that epilepsy associated with *Fgf9* deletion is closely related to a decrease in GABA.

Moreover, 128 epileptic patients were classified into focal seizure (41.41%), generalized seizure (5.47%), combined generalized and focal seizure (10.16%), and unknown group (42.97%) using the operational classification of seizure types by ILAE classification. The expression level of FGF9 in the serum of patients with focal seizures was significantly lower than that in controls (*P* value < 0.0001). The ROC analysis also indicated that FGF9 had a diagnostic efficiency for focal seizures with an AUC of 0.725 (Fig. 6G). Future studies need to be performed on other large-scale clinical specimens.

To the best of our knowledge, this is the first work to show that FGF9 plays essential roles in epilepsy pathology, and FGF9 expression is significantly lower in serum of epileptic patients, especially with focal seizures. FGF9 plays a critical role in promoting the survival of GABAergic neurons and maintaining the physiological level of GABA, and appears to be a new candidate gene for epileptogenesis.

## Materials and methods

### Mice

*Fgf9*^*fl/fl*^ mice were obtained from Biocytogene (Beijing, China). *Fgf9*^*fl/fl*^ mice had floxp sites inserted across exon 1 of the target gene in the C57BL/6 background by a conventional gene-targeting strategy. To generate *Fgf9 CKO* mice, we crossed *Fgf9*^*fl/fl*^ mice with *Olig1-Cre*, *Olig2-Cre*, *Nestin-Cre*, *VGAT-Cre*, *VGLUT1-Cre*, *Chat-Cre*, *GFAP-Cre*, *DAT-Cre*, and *Mpz-Cre* mice (Jackson Laboratory). Mice were genotyped by PCR analysis (Table S4) of DNA obtained from tail tissue. Mice were maintained in the specific pathogen-free facility of the Animal Center of Second Hospital of Hebei Medical University. All experiments were conducted in accordance with protocols approved by the Institutional Animal Care and Use Committee of Second Hospital of Hebei Medical University (2020-R155).

### Antibodies and chemicals

Primary antibodies targeting the following proteins were used for western blot and tissue sections: FGF9 (Abcam, EPR19937), Olig1 (Millipore, MAB5540), NeuN (Millipore, ABN78), Iba-1 (Abcam, ab5076), anti-GFAP (Millipore, MAB360), anti-Calbindin (Sigma, C2724), Caspase-3 (Santa Cruz, sc-373730), GABA (Sigma, A2052), VGAT (SYSY, 131011), Calbindin (Sigma, C2724), GAPDH (Abcam,ab128915), Adcy5/6 (Santa Cruz, sc-514785), p-ERK (Cell Signaling, #4370), ERK (Cell Signaling, #4695), p-Akt (Immunoway, YP006), Akt (Immunoway, YT0176), and β-actin (Proteintech, 60008-1-Ig). Secondary antibodies for western blot were from Rockland Immunochemicals, USA, and fluorescein isothiocyanate secondary antibodies were from Jackson ImmunoResearch, West Grove, PA. Hoechst stain (1:100, Invitrogen), RNeasy Lipid Tissue Mini kit (QIAGEN, 74804), SQ22536, bupivacaine-HCl, pentylenetetrazol, GABA, and Glu were purchased from Sigma (Madrid, Spain).

### EEG video

The mice were implanted with EEG electrodes at approximately 2 months of age. All the implanted mice were anesthetized with chloral hydrate (5%), the skull was exposed, and electrodes were implanted on the surface of the cortex (1 mm lateral to midline, 2.0 mm posterior to bregma). A ground electrode was implanted on the cerebellum (at midline, 2.0 mm posterior to lambda). The parameters of EEG recording were as follows: high-pass filtering, 1 Hz; low-pass filtering, 100 Hz; gain, 1000 times; and sampling frequency, 1 k/s. EEG signals were analyzed by two individuals who were blind to the experimental condition, and the behavior was monitored by video.

### UHPLC-MS/MS

The measurement of GABA and Glu was performed as described by Gonzalez et al^44^. The instrument was operated using an electrospray ionization source (ESI) in positive mode. ESI parameters were capillary voltage, 3.0 kV; extractor voltage, 3 V; source temperature, 120 °C; desolvation temperature, 350 °C; cone gas flow, 80 L/h; and desolvation flow, 600 L/h (both gases were nitrogen).

### Immunohistochemistry and immunofluorescence

The mice were sacrificed and perfused with 4% paraformaldehyde. Immunofluorescence and immunohistochemistry staining were performed as described previously, with some modifications^45^. For immunofluorescence staining, the brain sections were probed with a primary antibody at 4 °C overnight and with a secondary antibody and Hoechst stain for 1 h in a light-resistant container at room temperature. Images were obtained using a fluorescence confocal microscope (Olympus FV1000). For immunohistochemistry staining, the sections were incubated with a primary antibody at 4 °C overnight and then separately incubated with a biotinylated secondary antibody and horseradish peroxidase (HRP)-streptavidin at room temperature for 30 min, followed by staining with 3, 3′-diaminobenzidine. Images were captured using an Olympus BX51 microscope and a DP72 digital camera.

### RNA-seq and qRT-PCR

Total RNA was extracted from the cortex using TriPure Isolation Reagent (Roche) following the manufacturer’s protocol, and quantified using the NanoDrop 2000 (Thermo) and Agilent Bioanalyzer 2100 (Agilent). Then Oligo (dT) magnetic beads are used to select poly (A) + mRNA, which was used to synthesis cDNA via reverse transcription. The cDNA was amplified and subjected to 50-bp single-end sequencing with a BGISEQ-500 sequencer^46^. At least 20 million clean reads of sequencing depth were obtained for each sample. The DEGs were defined as genes with a false discovery rate (FDR) less than 0.01, and log2 fold-change greater than 1 (upregulation) or less than −1 (downregulation). The DEGs were screened after gene annotation and normalization, and then analyzed by including: Gene Ontology (GO) enrichment analysis, KEGG pathway enrichment analysis, cluster analysis, and protein–protein interaction network analysis. We performed qPCR using gene-specific primer sets (Table S3), and we assessed the relative gene expression (in triplicate) after normalization using a reference gene (GAPDH).

### Western blot analysis

Tissues were harvested from the sacrificed mice and transferred to tubes. Proteins were extracted using a protein extraction kit (Applygen Technologies Inc., P1250), separated by SDS-PAGE and then transferred to PVDF membranes. Membranes were incubated with primary antibodies for 12 h at 4 °C, washed 3 times for 15 min, and incubated with secondary antibodies for 1 h at 37 °C. Finally, membranes were scanned with an Odyssey Infrared Imaging System (LI-COR, Lincoln, NE).

### Drug and inhibitor administration

The *CKO*^*VGAT*^ and *CKO*^*Olig1*^ mice were observed by video, and the number of seizures within 48 h were counted. All the drugs and inhibitors were dissolved in 0.9% sodium chloride and administered intraperitoneally in a volume of 0.1 ml (gabapentin, 100 mg/kg body weight; SQ22536, 10 mg/kg; bupivacaine-HCl, 10 mg/kg) every 12 h. The number of seizures within 48 h after administration were counted. The *CKO*^*VGAT*^ mice were intranasally administered with rhFGF9 proteoliposomes (conc. 500 μg/kg) every 12 h, and the number of seizures within 72 h before and after administration were counted. The rhFGF9 proteoliposomes were gifted by Wenzhou Medical University.

### Pentylenetetrazol-induced mice

C57BL/6 J male mice (6 weeks) were randomly allocated into 2 groups: the control and pentylenetetrazol-induced model group (intraperitoneal injection of PTZ at a dose of 35 mg/kg). Kindling was induced on every alternate day, and mice were observed and seizure scores were recorded^47^. After attaining a seizure score of 4 (clonic seizures with loss of righting reflex) on 3 consecutive days, the mice were considered kindled, and then blood samples and brains were collected for further studies.

### Human serum

A total of 128 epileptic patients and 128 controls matched by age and sex were recruited from the Department of Neurology, Second Hospital of Hebei Medical University. The clinical examinations of the controls showed normal results. The research involving serum from control subjects and epilepsy patients was approved by the human research committee of Second Hospital of Hebei Medical University, and written informed consent was obtained from all participants before the study.

Blood samples (5 ml) were extracted from epileptic patients and healthy controls. Then, all the samples were centrifuged for 10 min at 2500 rpm, and the supernatant was stored at −80 °C until use. The human FGF9 ELISA kit (EIA-1448H), human GABA ELISA kit (EIA-0781), mouse FGF9 ELISA kit (EIA-2517M), and mouse GABA ELISA kit(EIA-2439) were purchased from Shanghai Elisa Biotech Co., Ltd, China (http://www.elsbiotech.cn/). The clinicopathologic characteristics of each patient were recorded in a database.

### Statistical analyses

All statistical analyses were carried out with SPSS 16.0 software, and the figures were designed by GraphPad Prism 5 and Adobe Illustrator CS6. Statistical significance in drug and inhibitor experiments, and qPCR and western blot analyses was calculated by paired Student’s *t*-test. In other experiments the variables with normal distributions were compared between two groups by using an independent samples *t*-test, and the Mann-Whitney *U* test was used to compare the non-normally distributed variables of two groups. Data are represented as mean ± SEM (error bars). *P* < 0.05 was considered to indicate statistical significance. *P* < 0.001 was considered to indicate high statistical significance.

## Supplementary information

Sup Figure caption.

Sup Fig 1.

Sup Fig 2.

Sup Fig 3.

Sup Fig 4.

Sup Fig 5.

Sup Fig 6.

Sup Table.

Legends for the Supplementary Video.

Sup video 1.

Sup video 2.

Sup video 3.

Sup video 4.

Sup video 5.

Sup video 6.

Sup video 7.
