# Supplementary material for: Deletion of FGF9 in GABAergic neurons causes epilepsy
Source: Cell Death Dis. 2021 Feb 19;12(2):196. doi: 10.1038/s41419-021-03478-1 (PMC7896082; doi:10.1038/s41419-021-03478-1)
Supplement: Supplementary file 1 — Sup Figure caption. [file 41419_2021_3478_MOESM1_ESM.docx]

Fig.S1 **There were no demyelination in the CNS of *CKO^Olig1^* mice.** Representative images of the CNS from *F/F* and *CKO^Olig1^* mice depicting the myelinated area (LFB). The staining degree of LFB in hippocampus (0.34±0.014 vs 0.35±0.016), medulla (0.24±0.004 vs 0.25±0.001), cervical cord (0.32±0.005 vs 0.030±0.009), lumbar myeloid (0.25±0.003 vs 0.24±0.001) in the cortex of *F/F* and *CKO^Olig1^* mice. n=3. Scale bar, 20 μm.

Fig.S2 **Immunohistochemical staining for astrocyte, microglia and neurons in the thalamus and hippocampus of *CKO^Olig1^* and *F/F* mice.** Scale bar, 20 μm.

Fig.S3 ***CKO^VGAT^* and *CKO^Nestin^* mice also show growth retardation phenotypes. (A and B)** *CKO^VGAT^* and *CKO^Nestin^* mice exhibited growth retardation, with a 40-30% lower body weight than *F/F* mice. Data are mean ± SEM for n=26 and n=20 mice per genotype. *P<0.05; **P<0.01, nonparametric Mann-Whitney test.

Fig.S4 **Glu and GABA concentrations in the cortex of *CKO* and control mice**. **(A)** GABA and Glu levels in the cortex of the control and *CKO^VGAT^* mice. **(B)** GABA and Glu levels in the cortex of the control and *CKO^Olig1^* mice. Three animals per group.

Fig.S5 **Computational analysis of differentially regulated pathways between *CKO^Olig1^* and control mice. (A)** Venn chart of coexpressed genes between the samples of *CKO^Olig1^* and control mice. **(B)** Scatter plots of all expressed genes in the hippocampus of *CKO^Olig1^* and control mice. **(C)** [KEGG pathway](file:///H:\GMR\RNA-Seq_report\src\page\Methods.html#9) mapping showing changes in DEGs associated with the AC/cAMP pathway in *CKO^Olig1^* mice versus control mice.

Fig.S6 **Changes of FGF9 expression were related to epilepsy. (A)** Western blot analyses of FGF9 and Adcy5 expression in pentylenetetrazole-induced mice compared to that in controls. **(B and C)** FGF9 expression at the mRNA and protein levels is basically consistent with ELISA results. Twenty-one epileptic patients and controls were randomly selected for verification.
