# Supplementary figures and images for: Deletion of FGF9 in GABAergic neurons causes epilepsy
Source: Cell Death Dis. 2021 Feb 19;12(2):196. doi: 10.1038/s41419-021-03478-1 (PMC7896082; doi:10.1038/s41419-021-03478-1)

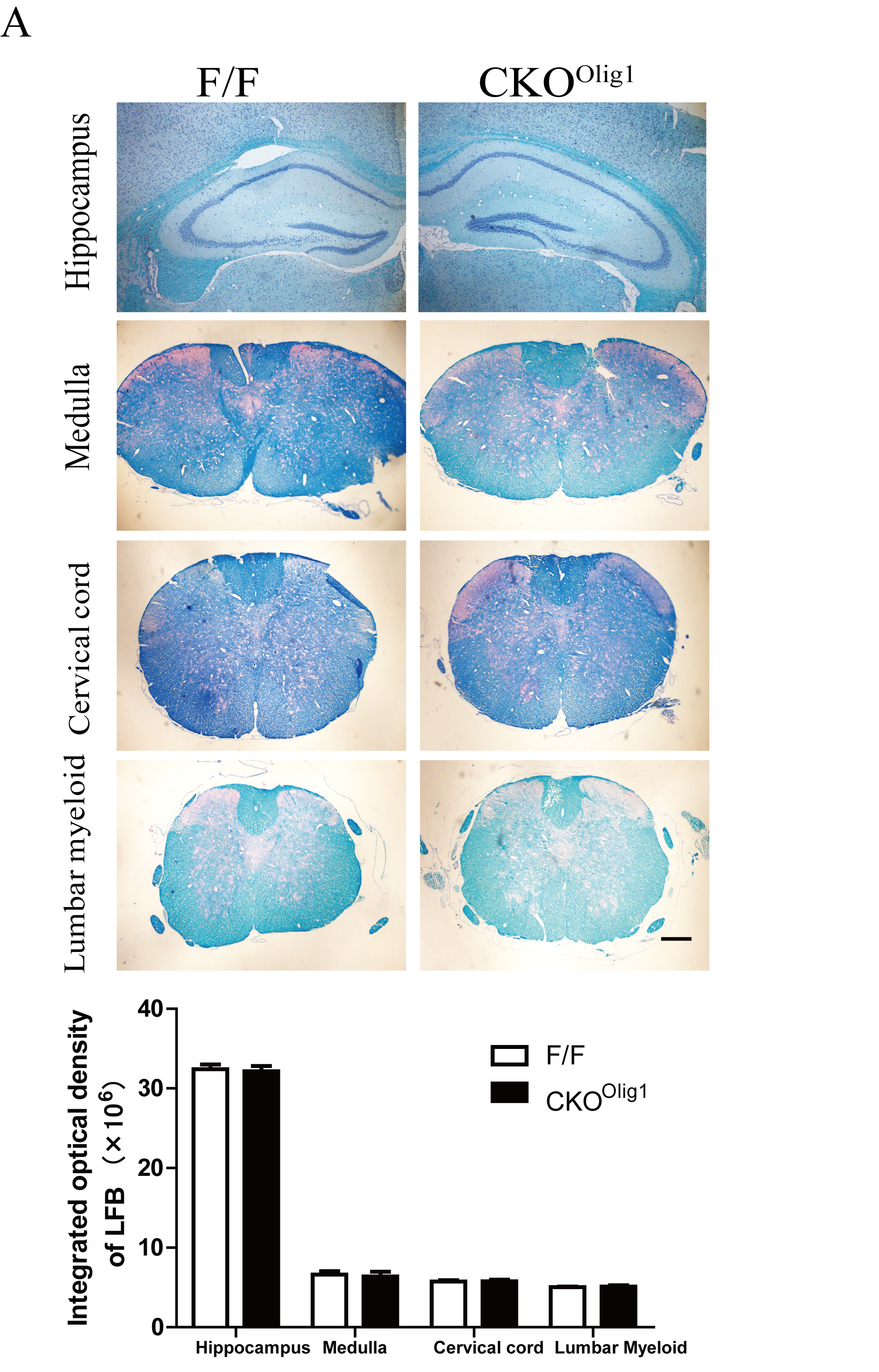

Supplement: Supplementary file 2 — Sup Fig 1. [file 41419_2021_3478_MOESM2_ESM.tif]

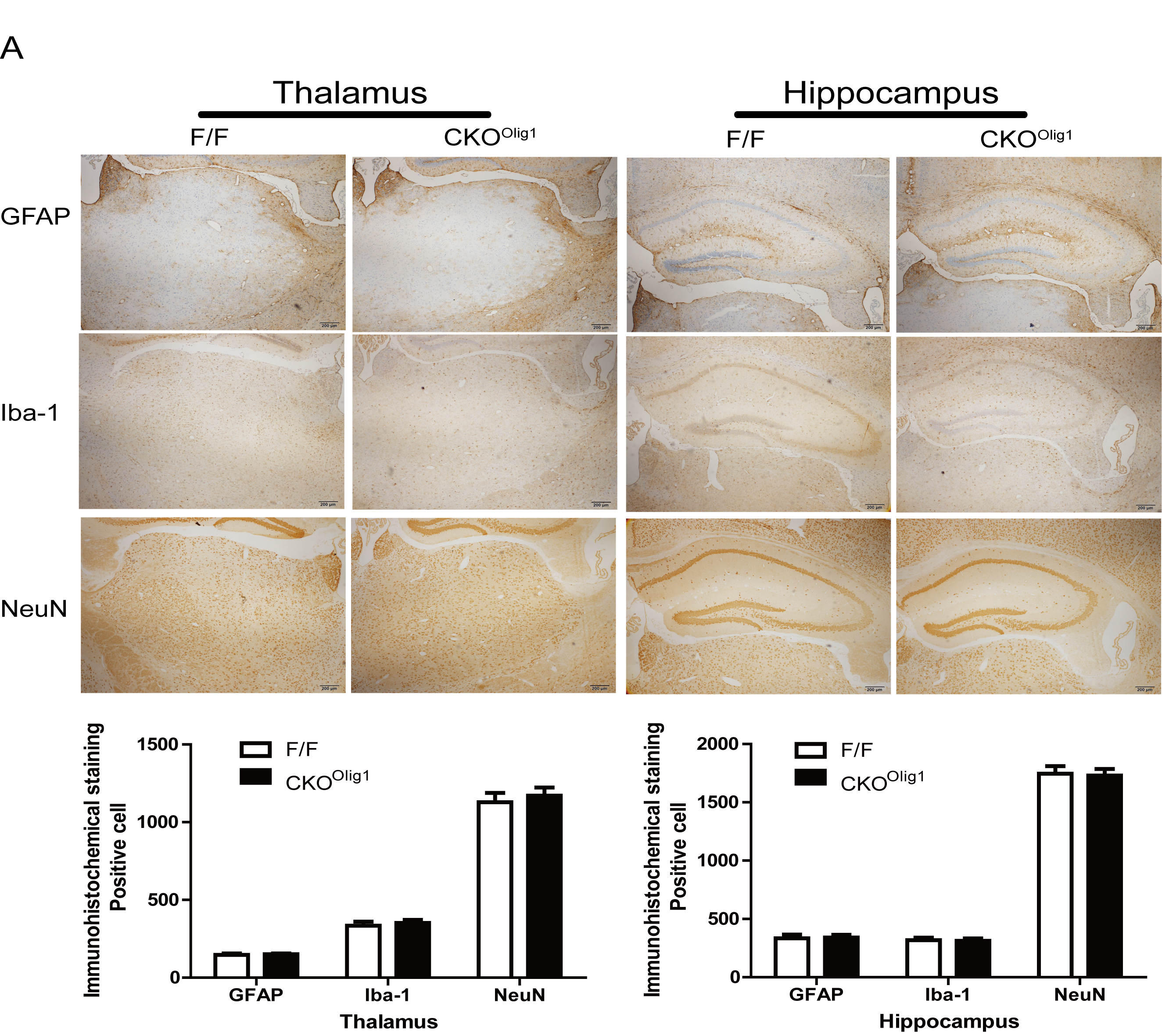

Supplement: Supplementary file 3 — Sup Fig 2. [file 41419_2021_3478_MOESM3_ESM.tif]

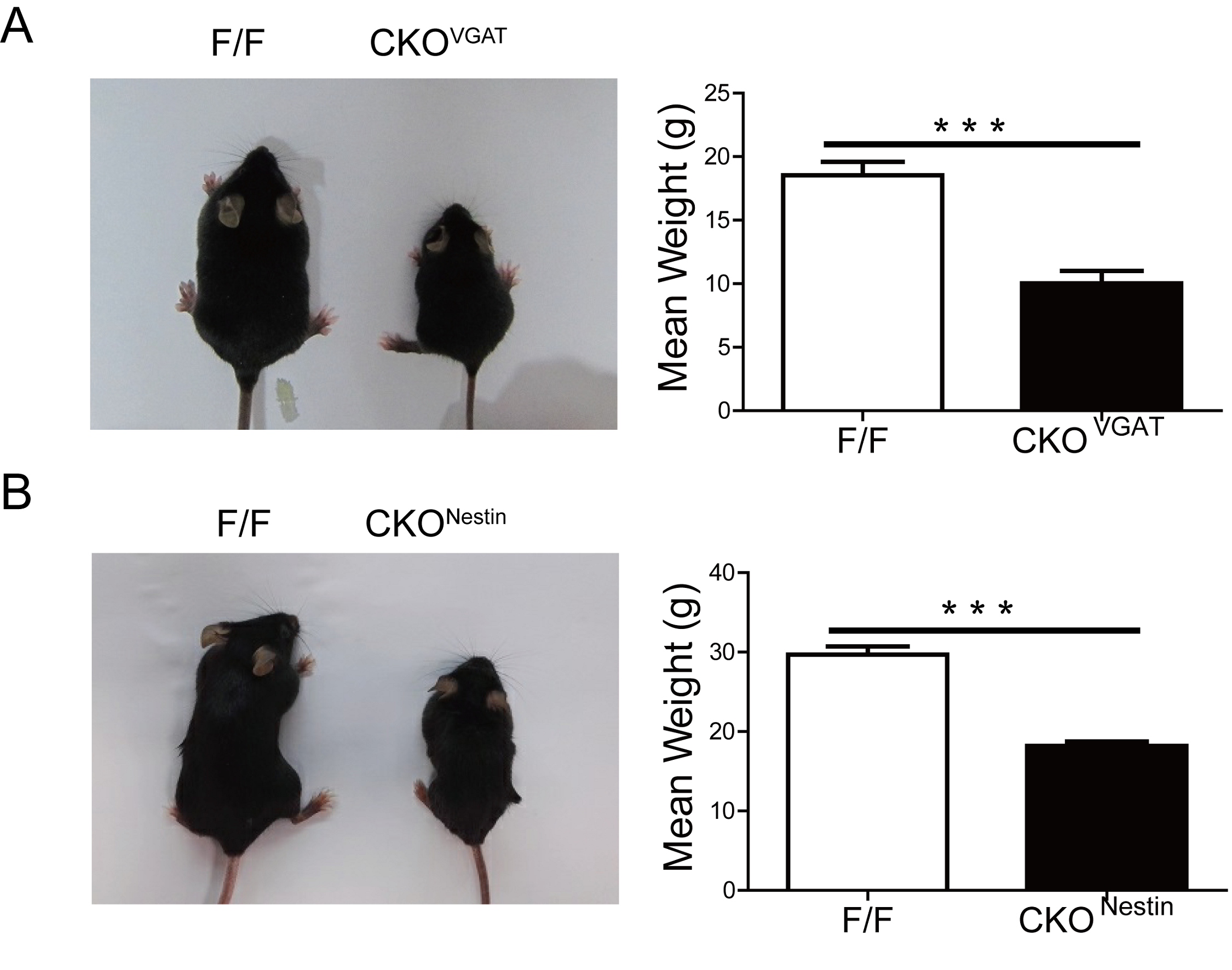

Supplement: Supplementary file 4 — Sup Fig 3. [file 41419_2021_3478_MOESM4_ESM.tif]

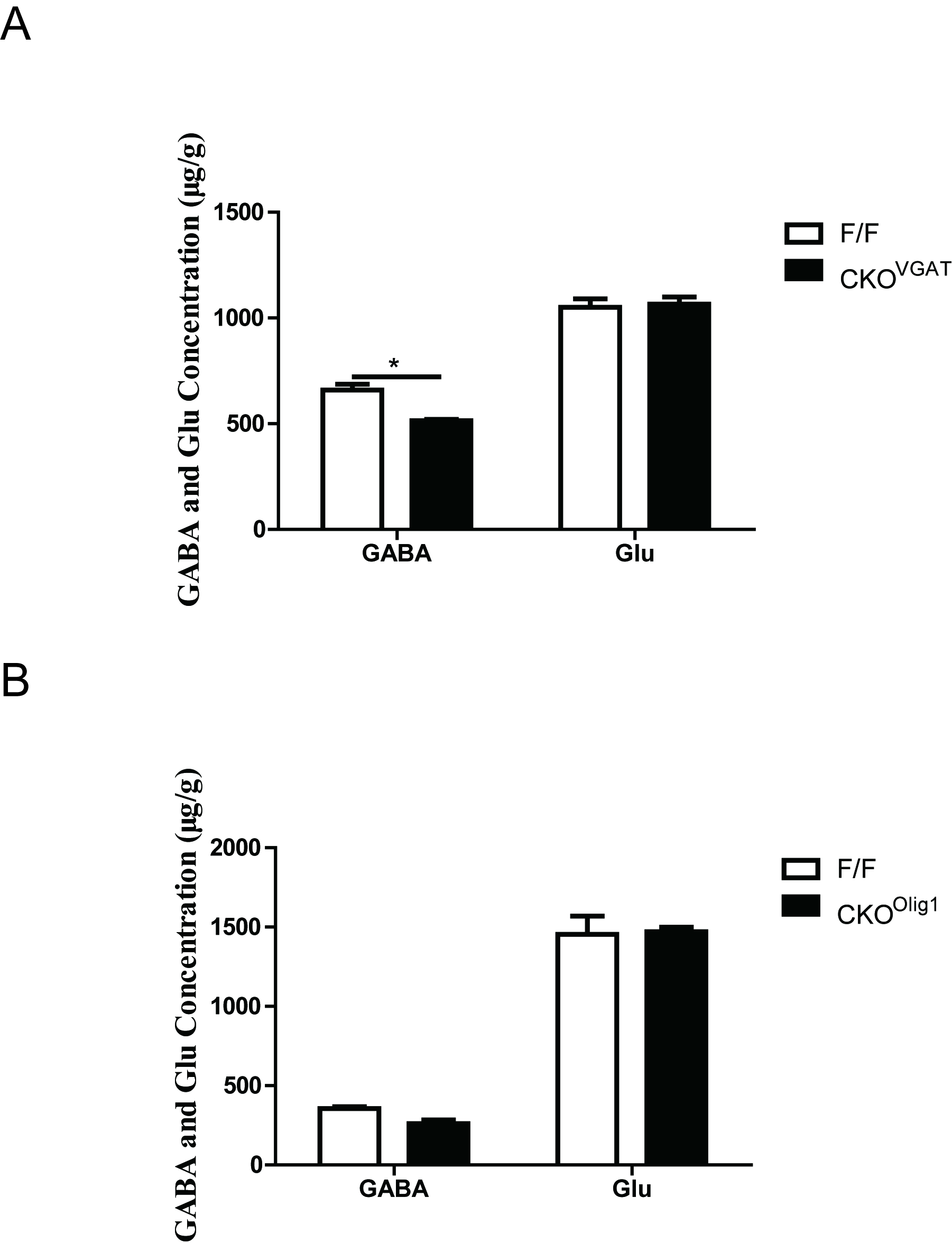

Supplement: Supplementary file 5 — Sup Fig 4. [file 41419_2021_3478_MOESM5_ESM.tif]

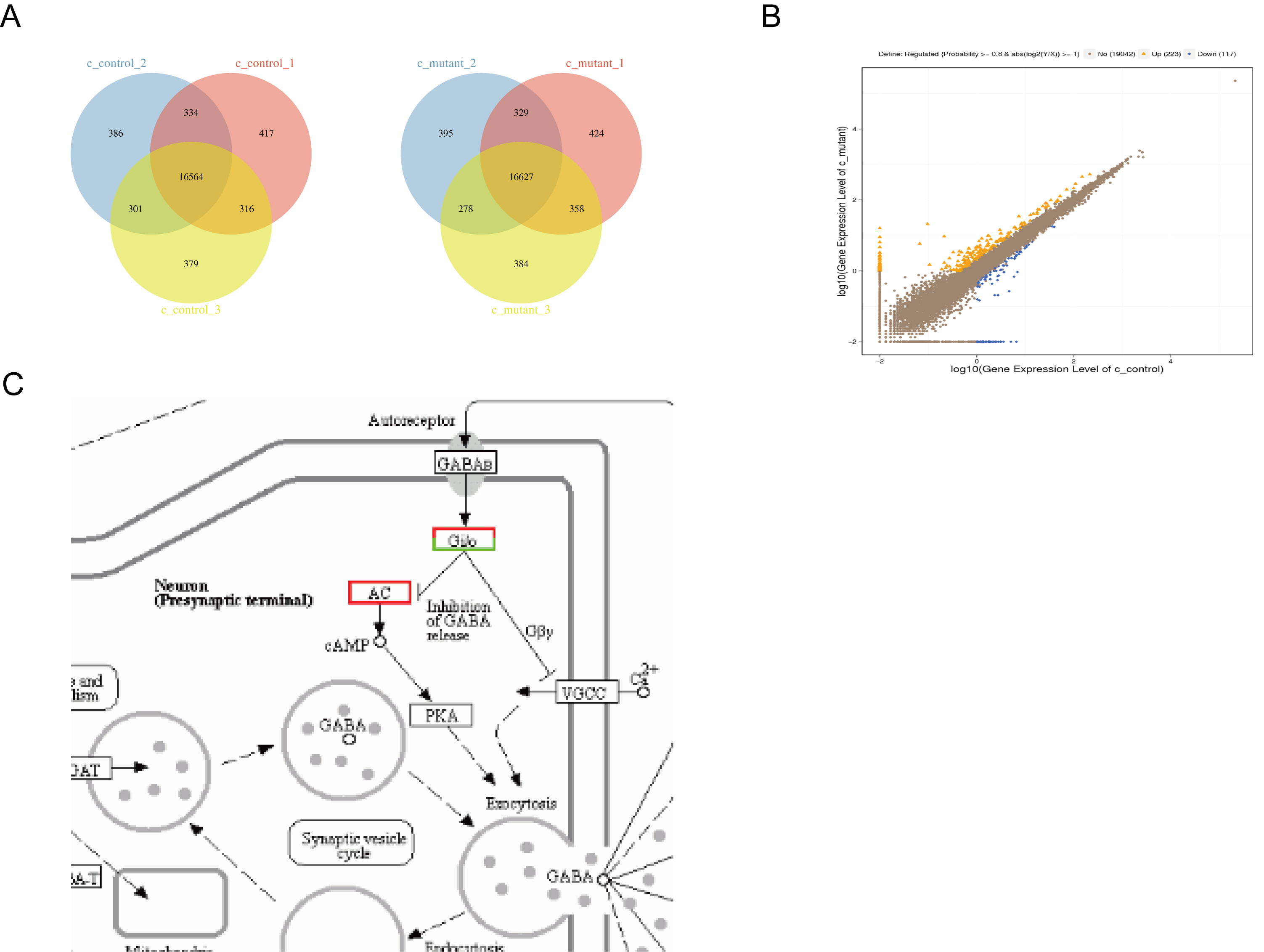

Supplement: Supplementary file 6 — Sup Fig 5. [file 41419_2021_3478_MOESM6_ESM.tif]

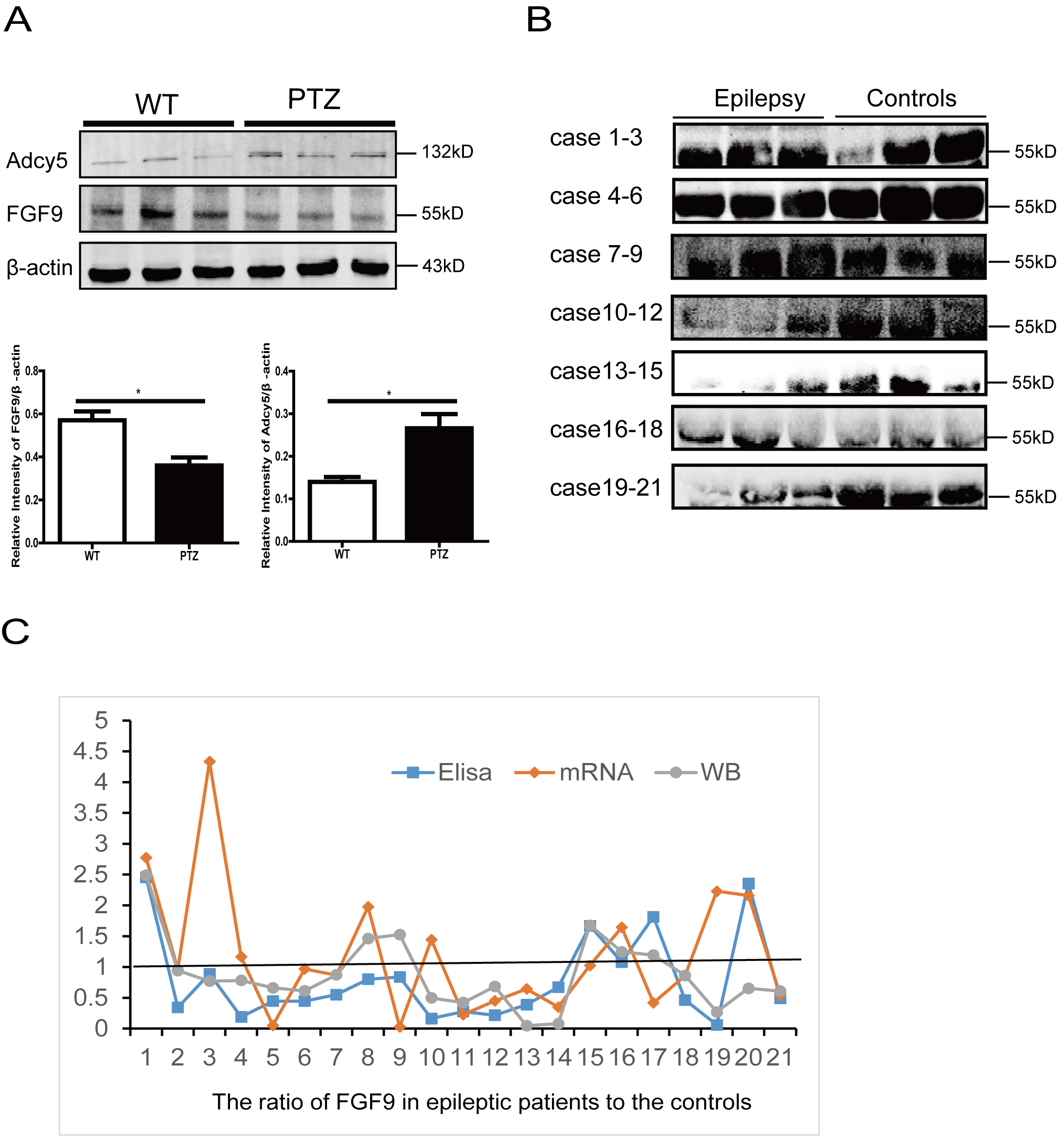

Supplement: Supplementary file 7 — Sup Fig 6. [file 41419_2021_3478_MOESM7_ESM.tif]
