# Supplementary material for: Deletion of FGF9 in GABAergic neurons causes epilepsy
Source: Cell Death Dis. 2021 Feb 19;12(2):196. doi: 10.1038/s41419-021-03478-1 (PMC7896082; doi:10.1038/s41419-021-03478-1)
Supplement: Supplementary file 8 — Sup Table. [file 41419_2021_3478_MOESM8_ESM.docx]

Table.S1 **Some genes were up-regulated in the *CKO^Olig1^* compared to *F/F*.**

| SN | Symbol | GeneID | log2Ratio(*CKO*/control) | Probability | Blast nr |
| --- | --- | --- | --- | --- | --- |
| 1 | Actn2 | 11472 | 1.315795983 | 0.986576747 | alpha-actinin-2 |
| 2 | Adcy5 | 224129 | 1.083910577 | 0.987581916 | adenylatecyclase type 5 |
| 3 | Agt | 11606 | 2.223460139 | 0.999214005 | angiotensinogen |
| 4 | Ano2 | 243634 | 1.15619089 | 0.986835072 | anoctamin-2 |
| 5 | Arhgap6 | 11856 | 1.570089224 | 0.984410959 | GTPase-activating protein 6 isoform c |
| 6 | Cd4 | 12504 | 1.290249137 | 0.991038255 | T-cell surface glycoprotein CD4 precursor |
| 7 | Cdhr1 | 170677 | 1.941732345 | 0.987949543 | protocadherin 21 |
| 8 | Chat | 12647 | 2.259259362 | 0.980703895 | choline O-acetyltransferase |
| 9 | Clic6 | 209195 | 2.709083813 | 0.997572995 | chloride intracellular channel protein 6 |
| 10 | Dlk1 | 13386 | 3.353956949 | 0.998812193 | protein delta homolog 1 isoform 1 precursor |
| 11 | Dlx6 | 13396 | 1.193628398 | 0.985705194 | homeobox protein DLX-6 |
| 12 | Doc2b | 13447 | 1.488551424 | 0.997506341 | double C2-like domain-containing protein beta |
| 13 | Drd1 | 13488 | 1.398549376 | 0.988277659 | D(1A) dopamine receptor |
| 14 | Ecel1 | 13599 | 2.247422339 | 0.999477761 | endothelin converting enzyme-like 1, isoform CRA_a |
| 15 | Fgf3 | 14174 | 2.534689294 | 0.999998232 | Fibroblast growth factor 3 |
| 16 | Filip1 | 70598 | 1.765070932 | 0.986374212 | filamin-A-interacting protein 1 |
| 17 | Gm9855 | 624784 | 6.445428759 | 0.99985232 | Thymine DNA glycosylase |
| 18 | Gng7 | 14708 | 1.377875708 | 0.999214589 | guanine nucleotide-binding protein G(I)(O) gamma-7 |
| 19 | Gpr52 | 620246 | 1.377457664 | 0.98134868 | probable G-protein coupled receptor 52 |
| 20 | Gpr6 | 140741 | 1.060076953 | 0.973685754 | G-protein coupled receptor 6 |
| 21 | Hap1 | 15114 | 1.249242439 | 0.9882431 | huntingtin-associated protein 1 isoform B |
| 22 | Kcna5 | 16493 | 1.420542605 | 0.981447092 | potassium voltage-gated channel subfamily A member 5 |
| 23 | Lhx8 | 16875 | 3.047589459 | 0.982964234 | LIM-homeodomain protein |
| 24 | Lrrc10b | 278795 | 1.617419229 | 0.993240483 | leucine-rich repeat-containing protein 10B |
| 25 | Necab2 | 117148 | 1.133129442 | 1 | N-terminal EF-hand calcium-binding protein 2 |
| 26 | Ngfr | 18053 | 3.021859434 | 0.977975201 | tumor necrosis factor receptor superfamily 16 precursor |
| 27 | Ntrk1 | 18211 | 2.774516309 | 0.985867341 | high affinity nerve growth factor receptor precursor |
| 28 | Nts | 67405 | 2.242190152 | 0.99954685 | neurotensin/neuromedin N precursor |
| 29 | Oprk1 | 18387 | 1.314022897 | 0.990707338 | opioid receptor, kappa 1, isoform CRA_b |
| 30 | Oxt | 18429 | 5.45532722 | 0.999373742 | oxytocin-neurophysin 1 preproprotein |
| 31 | Pde10a | 23984 | 1.34095102 | 0.9894741 | cAMP and cAMP-inhibited cGMP 3',5'-cyclic phosphodiesterase 10A isoform 1 |
| 32 | Penk | 18619 | 1.592128672 | 1 | proenkephalin-A precursor |
| 33 | Scn4b | 399548 | 1.025711697 | 0.991108045 | sodium channel subunit beta-4 precursor |
| 34 | Scube3 | 268935 | 1.646532128 | 0.979634435 | signal peptide, CUB and EGF-like domain-containing protein 3 precursor |
| 35 | Sncg | 20618 | 1.127250082 | 0.931023505 | synuclein, gamma |
| 36 | Syndig1l | 627191 | 1.539907639 | 0.999945186 | synapse differentiation-inducing gene protein 1-like |
| 37 | Trh | 22044 | 1.655683673 | 0.984935772 | thyrotropin releasing hormone preproprotein |
| 38 | Vrk1 | 22367 | 1.00396345 | 0.993316458 | vaccinia related kinase 1, isoform CRA_b |
| 39 | Xkrx | 331524 | 1 | 0.934331096 | XK-related protein 2 |
| 40 | Zfp114 | 232966 | 1.438210099 | 0.867735057 | zinc finger protein 114 |

Table.S2 **Some genes were down-regulated in the *CKO^Olig1^* compared to *F/F*.**

| SN | Symbol | GeneID | log2Ratio(CKO/control) | Probability | Blast nr |
| --- | --- | --- | --- | --- | --- |
| 1 | Apln | 30878 | -1.379465261 | 0.958693698 | apelin |
| 2 | Ccl17 | 20295 | -1.196573248 | 0.923414499 | CC chemokine ABCD-2 |
| 3 | Cd7 | 12516 | -1.584962501 | 0.914524 | T-cell antigen CD7 precursor |
| 4 | Cdh19 | 227485 | -1.4665678 | 0.939571705 | cadherin-19 precursor |
| 5 | Cideb | 12684 | -1.223696258 | 0.938459456 | cell death activator CIDE-B |
| 6 | Cplx3 | 235415 | -1.022858535 | 0.925284564 | complexin-3 precursor |
| 7 | Crh | 12918 | -1.09072317 | 0.930646113 | corticoliberinpreproprotein |
| 8 | Dio2 | 13371 | -1.195440458 | 0.962210825 | type II iodothyroninedeiodinase |
| 9 | Dmp1 | 13406 | -1.703606997 | 0.957669844 | dentin matrix acidic phosphoprotein 1 precursor |
| 10 | Gdpd3 | 68616 | -3.51340924 | 0.987807267 | glycerophosphodiesterphosphodiesterase domain-containing protein 3 |
| 11 | Gm13304 | 100504346 | -1.45050218 | 0.918258581 | beta chemokine Exodus-2 |
| 12 | Gng8 | 14709 | -1.300535801 | 0.94941442 | guanine nucleotide-binding protein G(I) (O) gamma-8 |
| 13 | Golt1a | 68338 | -1.521236903 | 0.944811606 | vesicle transport protein GOT1A |
| 14 | Grb7 | 14786 | -1.703606997 | 0.950719196 | growth factor receptor-bound protein 7 |
| 15 | Hkdc1 | 216019 | -1.292371951 | 0.966955768 | Hexokinase domain containing 1 |
| 16 | Il12a | 16159 | -1.063976589 | 0.937593838 | interleukin-12 subunit alpha isoform 2 precursor |
| 17 | Itgb4 | 192897 | -1.041528324 | 0.943498338 | integrin beta-4 isoform 2 precursor |
| 18 | Kcng4 | 66733 | -1.992137882 | 0.975487996 | potassium voltage-gated channel subfamily G member 4 |
| 19 | Kcnj10 | 16513 | -1.00254243 | 0.975923311 | Kcnj10 protein, partial |
| 20 | Klhdc7a | 242721 | -1.067114196 | 0.924654323 | kelch domain-containing protein 7A |
| 21 | Lamc2 | 16782 | -1.312342046 | 0.867468723 | laminin subunit gamma-2 precursor |
| 22 | Layn | 244864 | -1.010726407 | 0.940290331 | layilin precursor |
| 23 | Ly96 | 17087 | -1.220940287 | 0.92890201 | lymphocyte antigen 96 isoform A precursor |
| 24 | Mylk3 | 213435 | -1.521397372 | 0.942742856 | myosin light chain kinase 3 isoform 2 |
| 25 | Napsa | 16541 | -1.03542949 | 0.873671854 | napsin-A precursor |
| 26 | Nat8f3 | 93674 | -1.169517057 | 0.949675848 | probable N-acetyltransferase CML3 |
| 27 | Ninj2 | 29862 | -1.25022663 | 0.937295247 | ninjurin-2 |
| 28 | Ovol2 | 107586 | -1.173040302 | 0.966868514 | zinc finger OVO2 isoform A |
| 29 | Plb1 | 665270 | -1.266786541 | 0.883982086 | phospholipase B1 |
| 30 | Prph | 19132 | -1.136980903 | 0.878591861 | peripherin isoform 1 |
| 31 | Prtn3 | 19152 | -1.404903122 | 0.952372226 | myeloblastin precursor |
| 32 | Rnf39 | 386454 | -1.331038513 | 0.950054549 | /RING finger protein 39 |
| 33 | Rrm2 | 20135 | -1.028196892 | 0.905807681 | ribonucleotidereductase M2 |
| 34 | Snap25 | [12477932](https://www.ncbi.nlm.nih.gov/pubmed/12477932) | -2.721535554 | 0.94323743 | Snap25 protein |
| 35 | Sebox | 18292 | -1.343073904 | 0.943297179 | /homeobox protein SEBOX |
| 36 | Serinc2 | 230779 | -1.255210189 | 0.968394386 | unnamed protein product |
| 37 | Thbs4 | 21828 | -1.148098639 | 0.942871437 | thrombospondin-4 precursor |
| 38 | Tmsb15l | 399591 | -1.144491834 | 0.929368255 | RIKEN cDNA 4930488E11 |
| 39 | Trim12a | 76681 | -1.083901258 | 0.931887524 | Trim12 protein |
| 40 | Xdh | 22436 | -1.169925001 | 0.949577247 | xanthine dehydrogenase, isoform CRA_a |

Table.S3 **Group of qRT-PCR primers used to amplify gene-specific regions**

| **Gene name** | **Accession name** | | **Forward primer (5’-3’)** | **Reverse primer (5’-3’)** | **Tm (℃)** |
| --- | --- | --- | --- | --- | --- |
| Gng7 | NP_001033744.1 | CGTCTGACCTCATGAGCTACTGTGA | | CAAGGTTTCTTGTCCTTAAAGGGGTTC | 60 |
| Gng8 | XP_002722922.1 | CTGTCCCATTTTCCGTCCT | | CATCGCTTGCTTGCCTCGC | 60 |
| Adcy5 | NP_001012783.3 | GCTAGAGGCCAACAATGAGG | | GCCATGTAGGTGCTGCCTAT | 59 |
| Gdpd3 | NP_077190.2 | GGGTCAGACCGGCACATG | | CATGGGAGTCCTTGGAAATTTC | 61 |
| SNAP25 | AAH18249.1 | AGAACGCCCAGAGGAAGAG | | CAGGATTAGGGGAACACGC | 58 |
| Cplx3 | NP_666335.1 | CAGTTCACACAGAGGAAGGCAG | | CTCCTCGATCATCTTGGCTAGC | 60 |
| Ngfr | NP_150086.2 | GGAGAGAAACTGCACAGCGACA | | CAGGCTACTGTAGAGGTTGCCA | 61 |
| GAPDH | AAU89484.1 | GTCTCCTGCGACTTCAACAGCA | | ACCACCCTGTTGCTGTAGCCGT | 62 |

Table.S4 **Group of PCR primers used to amplify gene-specific regions in conditional knockout mice.**

| **Mice** | **Stock No** | **Forward primer (5’-3’)** | **Reverse primer (5’-3’)** | **Tm (℃)** |
| --- | --- | --- | --- | --- |
| FGF9^fl/fl^ |  | CTGCAGCGTTCTGGATACTAGCTT | GCTACGTCAGAATTTACGGATCCTGAC | 61 |
| Olig1-cre | 011105 | AAT CGC GAA CAT CTT CAG GT | CGC CCC AGA TGT ACT ATG C | 60 |
| Olig2-cre | 25567 | TTA CGG CGC TAA GGA TGA CT | CTT TCT TGG TGG AAG ACG TG | 60 |
| Nestin-cre | 003771 | GCG GTC TGG CAG TAA AAA CTA TC | GTG AAA CAG CAT TGC TGT CAC TT | 60 |
| Chat-cre | 018957 | CAA AAG CGC TCT GAA GTT CCT | CAG GGT TAG TAG GGG CTG AC | 60 |
| VGAT-cre | 017535 | GCA TTT CTG GGG ATT GCT TA | GTC ATC CTT AGC GCC GTA AA | 60 |
| VGLUT1-cre | 023527 | CCC TAG GAA TGC TCG TCA AG | ATG AGC GAG GAG AAG TGT GG | 60 |
| GFAP-cre | 012886 | TCC ATA AAG GCC CTG ACA TC | TGC GAA CCT CAT CAC TCG T | 60 |
| Mpz-cre | 017927 | CCA CCA CCT CTC CAT TGC AC | ATG TTT AGC TGG CCC AAA TG | 60 |
| DAT-cre | 006660 | TGG CTG TTG GTG TAA AGT GG | CCA AAA GAC GGC AAT ATG GT | 60 |
