# Supplementary material for: Deletion of FGF9 in GABAergic neurons causes epilepsy
Source: Cell Death Dis. 2021 Feb 19;12(2):196. doi: 10.1038/s41419-021-03478-1 (PMC7896082; doi:10.1038/s41419-021-03478-1)
Supplement: Supplementary file 9 — Legends for the Supplementary Video. [file 41419_2021_3478_MOESM9_ESM.pdf]

Sup Video.1 **The video of epilepsy in conditional knockout of *Fgf9* in *Olig1-cre* mice.**

Sup Video.2 **The video of epilepsy in conditional knockout of *Fgf9* in *Nestin-cre* mice.**

Sup Video.3 **The video of epilepsy in conditional knockout of *Fgf9* in *VGAT-cre* mice.**

Sup Video.4 **The video of epilepsy in conditional knockout of *Fgf9* in *VGLUT1-cre* mice.**

Sup Video.5 **The EEG-Video recordings for epileptic seizure duration in CKO^{VGAT} mice.**

Sup Video.6 **The EEG-Video recordings for interseizure interval in CKO^{VGAT} mice.**

Sup Video.6 **TheEEG-Video recordings of the control mice.**
